# *Arcanobacterium phocae* infection in mink (*Neovison vison*), seals (*Phoca vitulina*, *Halichoerus grypus*) and otters (*Lutra lutra*)

**DOI:** 10.1186/s13028-017-0342-8

**Published:** 2017-10-26

**Authors:** Bettina Nonnemann, Mariann Chriél, Gitte Larsen, Mette Sif Hansen, Elisabeth Holm, Karl Pedersen

**Affiliations:** 1 Department for Immunology and Vaccinology, National Veterinary Institute, Technical University of Denmark, Kemitorvet, Building 204, 2800 Kgs. Lyngby, Denmark; 2 Department for Diagnostics and Scientific Advice, National Veterinary Institute, Technical University of Denmark, Kemitorvet, Building 202, 2800 Kgs. Lyngby, Denmark

**Keywords:** *Arcanobacterium phocae*, *Arcanobacterium phocisimile*, Mink, Seal, Otter

## Abstract

**Background:**

Infectious skin disorders are not uncommon in mink. Such disorders are important as they have a negative impact on animal health and welfare as well as on the quality and value of the fur. This study presents the isolation of *Arcanobacterium phocae* from mink with severe skin lesions and other pathological conditions, and from wild seals and otters.

**Results:**

In 2015, *A. phocae* was isolated for the first time in Denmark from outbreaks of dermatitis in mink farms. The outbreaks affected at least 12 farms. Originating from these 12 farms, 23 animals cultured positive for *A. phocae*. The main clinical findings were necrotizing pododermatitis or dermatitis located to other body sites, such as the lumbar and cervical regions. *A. phocae* could be isolated from skin lesions and in nine animals also from liver, spleen and lung, indicating a systemic spread. The bacterium was also, for the first time in Denmark, detected in dead seals (n = 9) (lungs, throat or wounds) and otters (n = 2) (throat and foot).

**Conclusions:**

An infectious skin disorder in mink associated with *A. phocae* has started to occur in Danish farmed mink. The origin of the infection has not been identified and it is still not clear what the pathogenesis or the port of entry for *A. phocae* infections are.

**Electronic supplementary material:**

The online version of this article (doi:10.1186/s13028-017-0342-8) contains supplementary material, which is available to authorized users.

## Background

Infectious skin disorders are not uncommon in mink and are therefore of significance to the fur industry. Skin disorders have an impact on animal health and welfare and therefore also on the quality and value of the fur. Well-known skin disorders are the “sticky kit syndrome” and infected bite wounds [1, 2]. The bite wounds appear sporadically, although the brown mink show more aggressiveness than other breeds and therefore have an increased prevalence of bite wounds [1]. Secondary infections of bite wounds are often associated with bacteria like *Staphylococcus delphini* and *Streptococcus canis* [3]. These bacteria are also common in several other infectious conditions in mink, such as urinary tract infections, pneumonia, and pleuritis. In 1970 severe pyoderma was for the first time reported in mink in the USA and two decades later (i.e., the mid-1990s) also reported in Canada [4], but the condition had yet to be diagnosed in Scandinavia. The findings and causative agents were at the time believed to be a mixture of predisposed factors e.g., immune incompetence combined with secondary bacterial infections such as by staphylococci and streptococci [5]. In 2007 a similar type of skin infection, Fur Animal Epidemic Necrotic Pyoderma (FENP), was diagnosed in Finland [6]. Recently, Nordgren et al. [6] reported *Arcanobacterium phocae* as a potential causative pathogen of FENP commonly observed on the paws and facial skin. *A*. *phocae* is a Gram positive, non-motile, catalase positive, coryneform coccobacillus, which is beta-haemolytic on blood agar [7]. *A. phocae* was first isolated from seals and described as a new species in 1997 on the basis of biochemical and physiological characteristics supplemented with 16S rRNA phylogenetic analysis of the genus *Actinomyces* [8]. The hypothesized association between *A. phocae* in mink and seals is a historically use of seal meat as a source of high protein feed for farmed mink. Canadian mink farmers began using seal meat for mink in the mid to late 1990s, which coincided with the first reports of pododermatitis in Canadian mink [4]. FENP was also recently reported by the Danish Fur Farming Society [9]. This type of pyoderma tends to spread within and between farms causing poor animal health and economic loss [6]. We here report the first documented Danish cases of *A. phocae* associated pododermatitis and additionally the first cases of *A. phocae* without any association to pododermatitis.

## Methods

### Animals

**Fig. 1:**
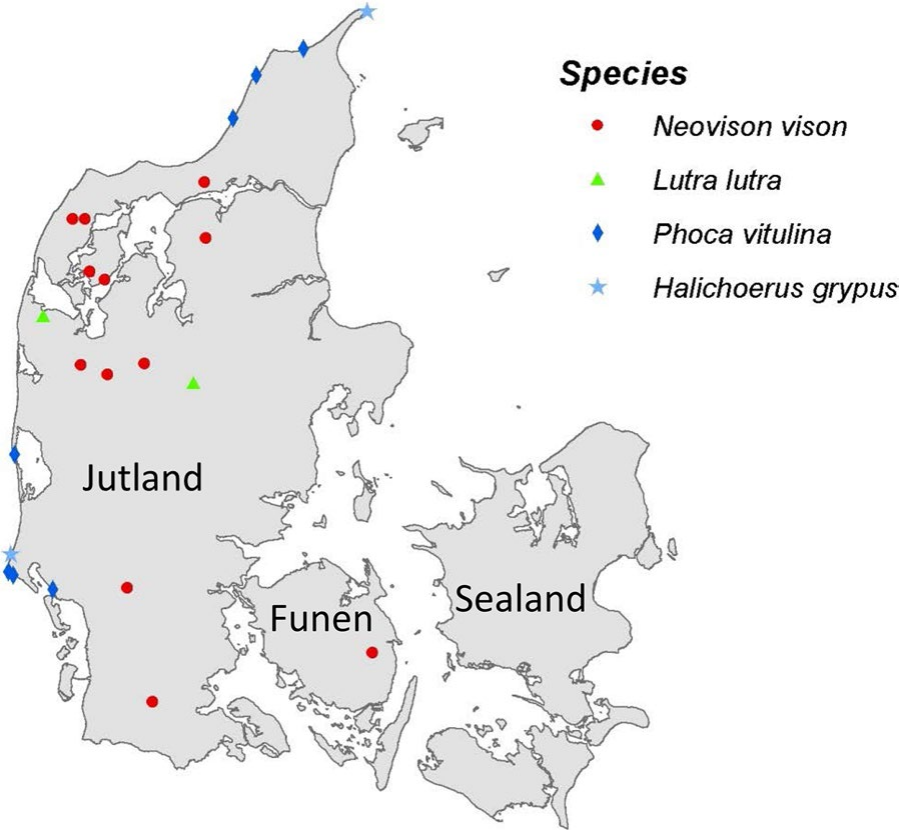
Distribution of mink, seals and otters infected with *Arcanobacterium phocae.* The majority of infected mink farms are located in Jutland and one farm is at the island of Funen. All seals were located on the coast of Jutland and otters were found in the countryside in Jutland

During the spring of 2015 till early winter 2015, 15 adult and eight juvenile mink (*Neovison vison*) carcasses, Nos. 1–23, (Table 1) originating from 12 mink farms (Fig. 1) were submitted to the National Veterinary Institute, Technical University of Denmark for laboratory examination. The animals were subjected to necropsy and follow up diagnostic examination including microbiological examination. Additionally, seven harbor seals (*Phoca vitulina,* Nos. 24–30), two grey seals (*Halichoerus grypus,* Nos. 31 and 32) and two otters (*Lutra lutra*, Nos. 33 and 34) were submitted to the laboratory during the fall/winter 2015/16. All submitted seals and otters were free-ranging animals, which were either found dead or had been euthanized due to animal welfare reasons.

**Table 1:** Isolation of *Arcanobacterium phocae* in mink, seals and otters

	Lung	Liver	Thoracic cavity	Nasal cavity/throat	Foot/flipper	Skin/ulcer
*Neovison vison (case no.)*	5, 6, 10, 12, 13	11	1, 7, 8	4, 20, 21	3, 14, 19	2, 9, 15, 16, 17, 18, 22, 23
*Phoca vitulina (case no.)*	24	29	–	25, 28	30	24, 26, 27
*Halichoerus grypus (case no.)*	–	–	–	31	32	–
*Lutra lutra (case no.)*	–	–	–	34	34	33

### Pathological examination

The carcasses of mink, seals and otters were subjected to standard necropsy procedures. Except for three mink (Nos. 1, 5 and 6), specimens of lung, liver, spleen, duodenum, ileum and kidney were sampled for histology. Additional samples were taken from other organs with lesions e.g., skin and/or feet. All tissue samples were fixed in 10% neutral buffered formalin, processed by routine methods for histology, embedded in paraffin wax and cut in 3–5 μm sections. The sections were mounted on conventional glass slides and stained with haematoxylin and eosin for histopathological examination [10]. Seals and otters were not examined by histology.

### Bacteriological examination

Material from skin and internal organs was collected for bacteriological examination. Columbia agar supplemented with 5% calf blood (SSI Diagnostica, Hillerød, Denmark) and Drigalski agar (SSI Diagnostica) were inoculated and incubated aerobically at 37 °C. For some of the samples, Columbia blood agar plates supplemented with colistin (25,000 units/mL), were used to prevent swarming growth of *Proteus* spp. and incubated at 37 °C in an atmosphere of 10% CO_2_. Plates were read after 16–20 h. In case of growth of pin-point colonies, the plates were reincubated and read again after another 24 h. All colonies of interest were subcultured on blood agar, where after they were identified by Matrix-assisted laser desorption/ionization mass spectrometry (MALDI-TOF MS). Mass spectra were obtained using an Autoflex Speed instrument (Bruker Daltonics, Bremen, Germany) calibrated with the Bruker *Escherichia coli* Bacterial Test Standard for Mass Spectrometry. Isolates were analysed, as described by Bizzini et al. [11], by the MALDI Biotyper RTC 3.1 software using a library of 6903 spectra using BDAL database (Bruker Daltonics) combined with verified local spectra from National Veterinary Institute. Included in the BDAL database are six different species of *Arcanobacteria* in which the species *A. phocae* is represented by 8 spectra and *Arcanobacterium phocisimile* is represented by 4 spectra. The MALDI Biotyper RTC 3.1 software compares the 10 closest spectra to the sample and provides a log score with a cut-off value of 2.0 for identification at species level and 1.7 for identification at genus level. These cut-off values were used as recommended by the manufacturer.

### Virological examination

Suspicion of influenza led to virological examinations in two mink (Nos. 20 and 21, Additional file 1). Lungs from mink and seals were tested by real time polymerase chain reaction (RT-PCR) for influenza virus using RNeasy Mini QIAcube Kit (Qiagen, Copenhagen, Denmark) as previously described [12]. Blood samples from otters and all mink, but Nos. 1, 3, 4 and 10, were tested for Aleutian mink disease virus (AMDV) antibodies by counter-current immunoelectrophoresis at Kopenhagen Diagnostic as described by Cho and Ingram [13]. Lung samples from seals and one otter were tested for canine distemper virus by an in-house immunofluorescence test or RT-PCR according to [14].

## Results

### Gross pathology and histopathology

Detailed information on the pathology of individual mink, seals and otters are available in Additional file 1.

**Fig. 2:**
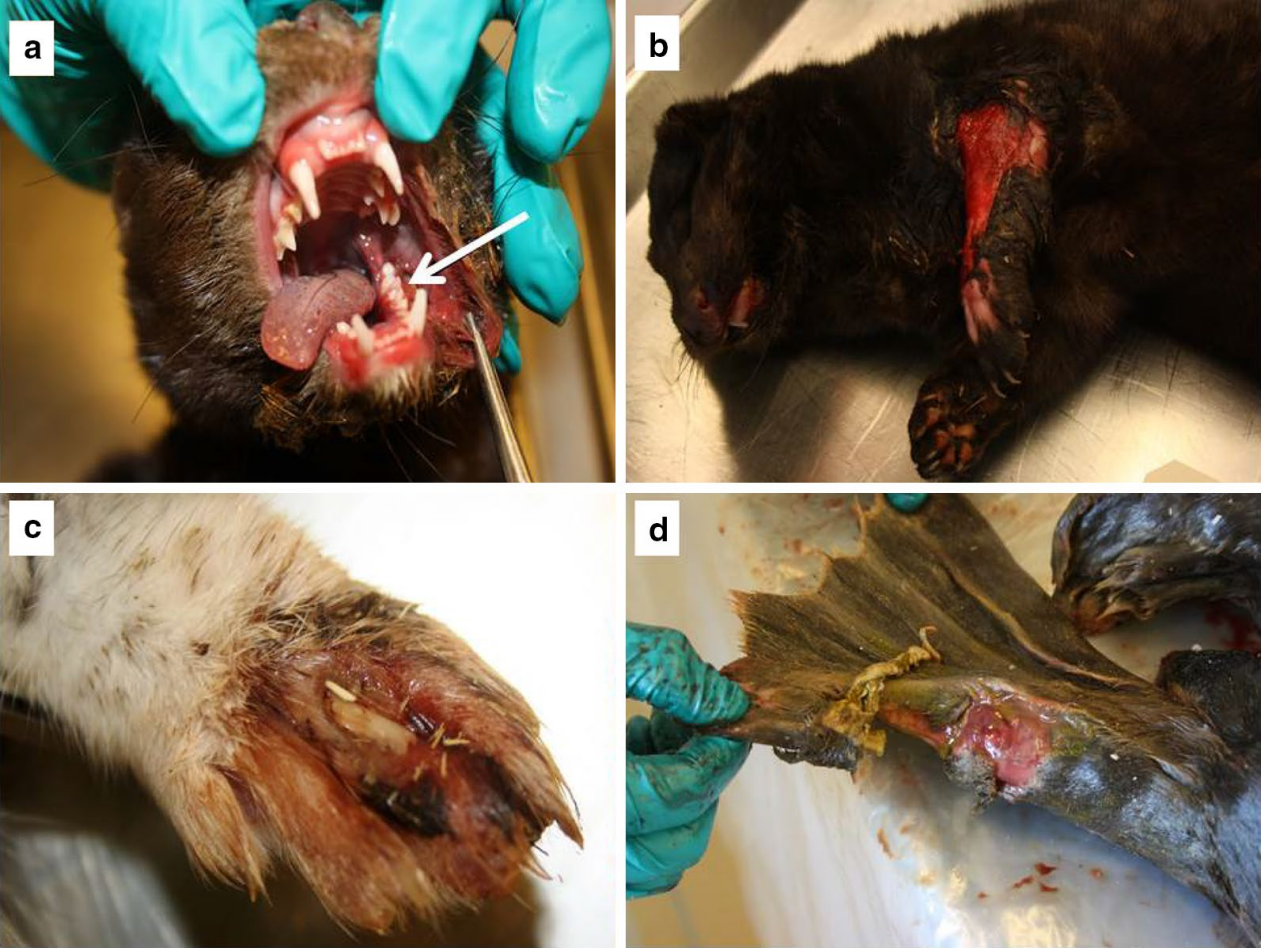
*Arcanobacterium phocae* associated lesions in mink and seals. **a** Mink with stomatitis of the buccal mucosa (arrow) and suppurative dermatitis of the cheek. *A. phocae* was cultured from the lesions. **b** Severe profound necrotizing dermatitis on the forelimb of a mink. *A. phocae* was cultured from pus. **c** Exudative pododermatitis caused by *A. phocae*. **d** Ulcer on rear flipper of a seal. *A. phocae* was cultured from the ulcer

In general, the mink carcasses were in good nutritional condition with moderate amounts of subcutaneous and abdominal fat. One mink was emaciated and three were obese. In total, 14 mink had skin lesions on the feet, legs, head and/or body, mainly represented by profound, suppurative and necrotizing pododermatitis on one or all feet (n = 10), some of which also had pustular to suppurative and necrotizing dermatitis on the legs (n = 4) or the head (n = 2) (Fig. 2). Other skin lesions were profound suppurative and necrotizing dermatitis in the head (n = 1) or in the lumbar region (n = 1), and dry crusts around nares and/or pus in the nasal cavity (n = 7) (Fig. 2). Six mink had an ulcer on the tip of the tail.

Pyothorax was a frequent finding (n = 8) often accompanied by pulmonary compression atelectasis. Other findings were suppurative bronchopneumonia and multifocal non-suppurative perivasculitis. Twelve mink had hepatomegaly due to congestion and most of these also had varying degrees of hepatocellular lipidosis. Furthermore, two mink had multifocal non-suppurative periportal hepatitis. Petechial haemorrhages were seen in the kidneys of five mink and two mink had focal or multifocal non-suppurative interstitial nephritis. Splenomegaly due to congestion was seen in 13 mink and petechial haemorrhages were seen in the spleen of four mink (Nos. 10, 11, 17 and 18). Furthermore, four mink had oral lesions such as fractured canine teeth, large amounts of dental calculus, stomatitis or gingival haemorrhages, and one pregnant mink had suppurative and necrotizing endometritis and placentitis. The findings in mink No. 22 with non-suppurative interstitial nephritis and non-suppurative periportal hepatitis, were consistent with Aleutian mink disease.

Five seals and one of the otters were emaciated, whereas the remaining seals and otter were in good body condition. Three seals had skin ulcerations (Fig. 2) and one otter had an abscess on the lower jaw. Most of the seals had lungworms (*Filaroides gymnurus* and *Otostrongylus circumlitus*) (n = 6) and one of these seals also had suppurative bronchopneumonia. Examination of the liver showed disseminated white pinpoint processes in one otter.

### Virological and serological examinations

Both mink tested for influenza virus were negative. Mink no. 22 tested positive for AMDV. All seals tested negative for influenza virus and canine distemper virus. Otters tested negative for AMDV and the otter, which was tested for canine distemper virus, was negative.

### Bacteriological examination

Bacteriological culture from skin lesions of mink revealed growth of pin-point, beta-haemolytic, white colonies, barely visible after 16–24 h of incubation, but clearly visible after 2 days of incubation. Many samples also displayed growth indicative of beta-haemolytic streptococci and haemolytic staphylococci. Colonies were subcultured and the monocultures subjected to identification by using MALDI-TOF. The pin-point colonies were identified as *A. phocae*, all with a log score above 2.0, while other pathogenic bacteria were identified as *S. canis* and *S. delphini*. *A. phocae* was not only isolated from skin lesions, but in some mink also from lung (n = 5), liver (n = 1), thoracic empyema (n = 3), and nose or nasal swabs (n = 3) (Table 1). In the seals and the two otters, bacteriological cultures from skin lesions, pharynx, lung and liver were also identified as *A. phocae* (Table 1, Additional file 1) while other findings were S. *canis*, *Streptococcus dysgalactiae* and *E. coli*. Notably, in one of the seals, *A*. *phocisimile* was found in an infected flipper.

## Discussion

*Arcanobacterium phocae* was for the first time in Denmark isolated from cases of dermatitis and other pathological conditions in mink, seals and otters. Furthermore the detection of *A. phocae* in nine seals and two otters shown here suggest the existence of a wildlife reservoir. The bacterium, *A. phocae*, originally isolated from lesions and internal organs in seals living in the coastal waters surrounding Scotland [7, 8], has in recent years become an important pathogen in farmed mink in both Europe [15] and Canada [3]. Hypothetically, since association with disease was originally observed in marine animals [4] and the tiny colonies could easily be overlooked, overgrown by contaminant flora, or mixed up with streptococci, *A. phocae* may have been present and associated with disease in mink earlier than reported. Prior to 1997 *A. phocae* had not been characterized and was therefore unknown as a pathogen. Historically, until the description of *A. phocae* as a pathogen, the bacterium has been suggested to have been misidentified as *Listeria ivanovii* in seals [7], which is known as a pathogen in ruminants and humans [16, 17]. In the genus *Arcanobacterium* another member, *A. phocisimile*, with close phenotypically resemblance to *A. phocae* was recently detected in seals from the German North Sea [18].

In the present study*, A. phocae* was identified in 23 farmed mink, 9 seals and 2 otters. The bacterium was isolated from both skin and internal organs (Table 1) and identified using the MALDI TOF technique. This technique has during the last decade proven to be a fast and reliable tool primarily used for diagnostics of humane pathogens. Recently, the technique has gained ground in in veterinary diagnostics in Scandinavia as well as other parts of northern Europe. The success of this technique relies on a well-equipped database containing suitable reference spectra for human and animal pathogens. A challenge for this method is however also the need for monocultures, which is a particular challenge when dealing with dermatitis due to the high risk of contamination or infection with secondary pathogens. In this study concurrent cultivation and identification of *S. canis*, *S*. *dysgalactiae*, *S*. *schleiferi* and *S. delphini* from the skin and nose (data not shown) in the majority of the mink were conducted. Also, isolation and identification of *A. phocosimile* from one of the Danish seals (No. 31) is reported here. *A. phocosimile* was identified at species level and in spite of close phenotypical resemblance to *A. phocae*, the MALDI Biotyper software 3.1 was able to distinguish between the two species [19].

This is the first report where *A. phocae* is associated with disease and death without signs of pododermatitis (Nos. 1, 6–8, 12, 13, 20 and 21) in mink. Some of the mink had been submitted for examination due to a suspicion of Aleutian mink disease and the veterinary practitioner informed of problems with pododermatitis in one specific farm; unfortunately, no animals were submitted for laboratory investigation at that time (Peter Vase Hansen, personal communication).

It is still not clear what the pathogenesis or the port of entry for *A. phocae* is. Pododermatitis is speculated to be a multifactorial disease and it is suggested that genetic or immune factors and age could make the animals more susceptible [20]. Many streptococci and staphylococci are known commensals of the skin but act as opportunistic pathogens in case an ulcus is formed, e.g. due to trauma. It is not known if there is an interaction between streptococci, staphylococci and *A. phocae* in the skin infections examined in this study, but the bacteria might act synergistically and thereby aggravate a skin lesion. Since we here present an alternative pathological expression of mink with no prior signs of pododermatis, further investigations are necessary to determine the point of entry and which predisposing factors are essential for *A. phocae* to establish a focus of infection. Since Denmark and other Scandinavian countries are not accustomed to use wild life animals as ingredients in mink feed, we consider wildlife as an unlikely source of infection. Likewise, as this bacterium has not been described from food producing animals or fish, it is not likely that the feed, which contains slaughter offal, was the source of infection.

## Conclusions

Necrotic dermatitis on the feet and skin of Danish mink was associated to infection by *A. phocae*, *Staphylococcus* spp. and *Streptococcus* spp. The presence of streptococci and staphylococci in such lesions has been reported previously, but *A. phocae*, seems to play a major role. The findings in some mink indicate that a systemic spread of *A. phocae* may develop even in mink without pododermatits.

## Additional file

**Additional file 1.** Data on individual animals regarding isolation of *Arcanobacterium phocae* and gross and microscopic pathological findings.

## Abbreviations

AMDV: Aleutian mink disease virus
FENP: Fur Animal Epidemic Necrotic Pyoderma
MALDI Biotyper RTC software: Matrix-assisted laser desorption/ionization Biotyper Real Time Classification software
MALDI-TOF: Matrix-assisted laser desorption/ionization time of flight
RT-PCR: real time polymerase chain reaction
16S rRNA: 16 Subunit ribosomal ribonucleic acid
