# Supplementary material for: Arcanobacterium phocae infection in mink (Neovison vison), seals (Phoca vitulina, Halichoerus grypus) and otters (Lutra lutra)
Source: Acta Vet Scand. 2017 Oct 26;59:74. doi: 10.1186/s13028-017-0342-8 (PMC5658911; doi:10.1186/s13028-017-0342-8)
Supplement: Supplementary file 1 — Additional file 1. Data on individual animals regarding isolation of Arcanobacterium phocae and gross and microscopic pathological findings. [file 13028_2017_342_MOESM1_ESM.docx]

**Additional file 1:** Data on individual animals regarding isolation of *Arcanobacterium phocae* and gross and microscopic pathological findings

| **Species** | **Case No.** | ***A. phocae* isolated from** | | | | | | **Pathological findings** |
| --- | --- | --- | --- | --- | --- | --- | --- | --- |
|  |  | **Lung** | **Liver** | **Thorax** | **Nasal cavity/**  **throat** | **Foot/**  **flipper** | **Skin/**  **ulcer** |  |
| *Neovison vison* | 1 |  |  | X |  |  |  | Ulcer on tip of tail. Pyothorax. Enlarged congested liver. Renal petechial haemorrhages. |
|  | 2 |  |  |  |  |  | X | Ulcer on tip of tail. Acute profound suppurative and necrotizing pododermatitis on two feet with complete involvement of one hind leg. Enlarged congested liver. |
|  | 3 |  |  |  |  | X |  | Ulcer on tip of tail. Chronic profound suppurative and necrotizing pododermatitis on two feet. Suppurative and necrotizing dermatitis on the neck. Splenomegaly. |
|  | 4 |  |  |  | X |  |  | Ulcer on tip of tail. Subacute suppurative and necrotizing dermatitis with dry crusts around nares and hyperkeratosis of the surrounding epidermis. Splenomegaly. |
|  | 5 | X |  |  |  |  |  | Ulcer on tip of tail. Pododermatitis on one foot. Dry crusts around nares. Pyothorax. Enlarged pale liver (lipidosis). Splenomegaly. |
|  | 6 | X |  |  |  |  |  | Splenomegaly. |
|  | 7 |  |  | X |  |  |  | Pyothorax. |
|  | 8 |  |  | X |  |  |  | Emaciated. Pyothorax. Slight hepatomegaly. Splenomegaly. |
|  | 9 |  |  |  |  |  | X | Subacute severe superficial suppurative and necrotizing pododermatitis on all feet with hyperkeratosis of the surrounding epidermis. Dry crusts around nares. Suppurative bronchopneumonia. Hepatomegaly (lipidosis). Large amounts of dental calculus. |
|  | 10 | X |  |  |  |  |  | Pyothorax and pulmonary compression atelectasis. Hepatomegaly (lipidosis). Petechial haemorrhages in spleen and kidneys. |
|  | 11 |  | X |  |  |  |  | Pyothorax and pulmonary compression atelectasis. Hepatomegaly (lipidosis). Petechial haemorrhages in spleen and kidneys. Mild suppurative enteritis. |
|  | 12 | X |  |  |  |  |  | Pyothorax and pulmonary compression atelectasis. Hepatomegaly (lipidosis). Splenomegaly. Petechial haemorrhages in kidneys. |
|  | 13 | X |  |  |  |  |  | Pyothorax and pulmonary compression atelectasis. Hepatomegaly (lipidosis). Splenomegaly. Petechial haemorrhages in kidneys. |
|  | 14 |  |  |  |  | X |  | Chronic profound suppurative and necrotizing pododermatitis on one foot with complete involvement of the leg. Suppurative bronchopneumonia. Mild hepatocellular lipidosis. Fractured maxillar canine teeth. |
|  | 15 |  |  |  |  |  | X | Ulcer on tip of tail. Chronic profound suppurative and necrotizing pododermatitis on all feet. Acute suppurative and necrotizing dermatitis on the head. Buccal stomatitis. |
|  | 16 |  |  |  |  |  | X | Chronic profound suppurative and necrotizing pododermatitis and interdigital hemorrhage on all feet. Phlegmon and acanthosis on one leg. Dry crusts around nares. Splenomegaly. |
|  | 17 |  |  |  |  |  | X | Chronic profound suppurative and necrotizing pododermatitis and interdigital haemorrhage on all feet. Focal suppurative dermatitis on one leg. Dry crusts around nares and eyes. Splenomegaly. |
|  | 18 |  |  |  |  |  | X | Phlegmone with overlaying subacute superficial suppurative and necrotizing pododermatitis, and interdigital haemorrhage on all feet. Dry crusts around nares. Hepatomegaly (lipidosis). Splenomegaly. |
|  | 19 |  |  |  |  | X |  | Suppurative pododermatitis on one foot. Multifocal non-suppurative pulmonary perivasculitis. Enlarged congested liver with multifocal non-suppurative periportal hepatitis. Splenomegaly. Multifocal non-suppurative interstitial nephritis. Diffuse non-suppurative enteritis. Gingival haemorrhage. |
|  | 20 |  |  |  | X |  |  | Multifocal non-suppurative pulmonary perivasculitis. Focal non-suppurative interstitial nephritis. Pus in the nasal cavity and dry crusts around nares. Pregnant with 5 fetuses, acute suppurative and necrotizing placentitis and endometritis. |
|  | 21 |  |  |  | X |  |  | Multifocal non-suppurative pulmonary perivasculitis. |
|  | 22 |  |  |  |  |  | X | Chronic profound suppurative and necrotizing dermatitis on the head. Hepatomegaly (lipidosis). Splenomegaly. |
|  | 23 |  |  |  |  |  | X | Chronic profound suppurative and necrotizing dermatitis in the lumbar region. Hepatomegaly (lipidosis). Splenomegaly. |
| *Phoca vitulina* | 24 | X |  |  |  |  | X | Emaciated. Ulcers on both rear flippers. Massive lungworm infection (*Filaroides gymnurus*, *Otostrongylus circumlitus*). |
|  | 25 |  |  |  | X |  |  | Emaciated. |
|  | 26 |  |  |  |  |  | X | Emaciated. Skin ulcer in neck region. Slight hepatomegaly. |
|  | 27 |  |  |  |  |  | X | Ulcer on rear flipper. Massive lungworm infection (*F. gymnurus*, *O. circumlitus*). |
|  | 28 |  |  |  | X |  |  | Massive lungworm infection (*F. gymnurus*, *O. circumlitus*). |
|  | 29 |  | X |  |  |  |  | Heart- (*Acanthocheilonema spirocauda*) and lung worm (*F. gymnurus*, *O. circumlitus*) infections. |
|  | 30 |  |  |  |  | X |  | Emaciated. Massive lung worm infection (*F. gymnurus*, *O. circumlitus*). |
| *Halichoerus grypus* | 31 |  |  |  | X |  |  | Emaciated. Subcutaneous oedema proximal to rear flippers |
|  | 32 |  |  |  |  | X |  | Lung worm infection (*F. gymnurus*, *O. circumlitus*) and suppurative bronchopneumonia. |
| *Lutra lutra* | 33 |  |  |  |  |  | X | Emaciated. Abscess in jaw region. Disseminated pinpoint liver processes. |
|  | 34 |  |  |  | X | X |  | Roadkill. |
